# Forfeited hepatogenesis program and increased embryonic stem cell traits in young hepatocellular carcinoma (HCC) comparing to elderly HCC

**DOI:** 10.1186/1471-2164-14-736

**Published:** 2013-10-26

**Authors:** Hsei-Wei Wang, Tsung-Han Hsieh, SSu-Yi Huang, Gar-Yang Chau, Chien-Yi Tung, Chien-Wei Su, Jaw-Ching Wu

**Affiliations:** 1 Institute of Microbiology and Immunology, National Yang-Ming University, Taipei, Taiwan; 2 Institute of Clinical Medicine, National Yang-Ming University, No. 201, Sec. 2, Shih-Pai Rd, Taipei, Taiwan; 3 Cancer Research Center & Genome Research Center, National Yang-Ming University, No. 201, Sec. 2, Shih-Pai Rd, Taipei, Taiwan; 4 Department of Education and Research, Taipei City Hospital, Taipei, Taiwan; 5 Division of General Surgery, Department of Surgery, Taipei Veterans General Hospital, Taipei, Taiwan; 6 Division of Gastroenterology, Department of Medicine, Taipei Veterans General Hospital, Taipei, Taiwan; 7 Department of Medical Research and Education, Taipei Veterans General Hospital, Taipei, Taiwan

**Keywords:** Young hepatocellular carcinoma, Embryonic stem cells, Dedifferentiation

## Abstract

**Background:**

Hepatocellular carcinoma (HCC) in young subjects is rare but more devastating. We hypothesize that genes and etiological pathways are unique to young HCC (yHCC; ≤40 years old at diagnosis) patients. We therefore compared the gene expression profiles between yHCCs and HCCs from elderly patients.

**Results:**

All 44 young HCCs (≤40 years old at the diagnosis; 23 cases in the training set while another 21 in the validation cohort) were positive for serum hepatitis B surface antigen (HBsAg), but negative for antibodies to hepatitis C virus (anti-HCV). All 48 elderly (>40 years old; 38 in the training set while another 10 in the validation cohort) HCC patients enrolled were also serum HBsAg positive and anti-HCV negative. Comparative genomics analysis was further performed for elucidating enriched or suppressed biological activities in different HCC subtypes.

The yHCC group showed more macroscopic venous invasions (60.9% vs. 10.5%, p < 0.001), fewer associated cirrhosis (17.4% vs. 63.2%, p < 0.001), and distinct profiles of expressed genes, especially those related to DNA replication and repair. yHCCs possessed increased embryonic stem cell (ESC) traits and were more dedifferentiated. A 309-gene signature was obtained from two training cohorts and validated in another independent data set. The ILF3 ESC gene, which was previously reported in poorly differentiated breast cancers and bladder carcinomas, was also present in yHCCs. Genes associated with HCC suppression, including AR and ADRA1A, were less abundant in yHCCs. ESC genes were also more enriched in advanced HCCs from elderly patients.

**Conclusion:**

This study revealed the molecular makeup of yHCC and the link between ESC traits and HCC subtypes. Findings in elderly tumors, therefore, cannot be simply extrapolated to young patients, and yHCC should be treated differently.

## Background

Hepatocellular carcinoma (HCC) is one of the most common cancers worldwide and chronic hepatitis B virus (HBV) infection is the most important cause of HCC in Taiwan [1,2]. Most HCC patients are diagnosed in old age with only a small portion of them younger than 40 years old [1,2]. Compared with the elderly HCC patients, young HCC (yHCC) cases (≤40 years of age) are more likely to be symptomatic at diagnosis and the HCC stage tends to be more advanced. Thus, there is a decreased chance of curative resection for the tumors in this group [3,4]. Although the presence of cirrhosis is less frequent in young patients [4], the time to yHCC recurrence after surgical resection was shorter and a one year survival rate was lower than those with elderly patients [4,5]. An aggressive clinical course and a poor prognosis have also been reported in children with HCC [6,7]. If yHCC patients survived longer than one year, their long-term survivals seemed to be better than those of elderly HCC patients due to fewer incidences of associated cirrhosis and relatively better liver function reserves [5]. High serum alpha-fetoprotein levels are more often found in yHCC patients [3,8]. HBV viral load is not a predictor in the development of HCC in young adults [9-11], in contrast, viral load and hepatic inflammatory activity were associated with late recurrence of HCC among elderly patients after resection of the primary HCC [12]. The aforementioned findings suggest that hepatocarcinogenesis in yHCCs is different from that in elderly patients. Yet the underlying mechanisms and the detail molecular portrait of yHCC remain unclear.

It has also been recognized that cancer cells, especially those of advanced and metastatic cancers, possess characteristics reminiscent of normal stem cells. The degree of stem cell gene reactivation or tumor cell dedifferentiation correlates with pivotal tumor features and prognosis [13,14]. A recent paper demonstrated by RT-qPCR, that the high expression levels of putative hepatic stem/progenitor cell biomarkers are related to tumor angiogenesis and a poor prognosis for HCC [15]. However, no similar study has addressed yHCC. Identifying genes involved in cancer progression and cell dedifferentiation offers another dimension to predict HCC recurrence, as well as providing novel therapeutic targets and prognosis markers.

## Results

### Clinical profiles, serological data, and histopathological findings for the HCCs from young and elderly patients enrolled in array analysis

The clinical profiles, serological data, and histopathological findings for young and elderly HCC patients in the training cohort are in Table 1. In 61 enrolled primary HBsAg positive HCC patients, 23 cases were yHCCs and 38 were elderly. Macroscopic venous invasion was more frequent (60.9% vs. 10.5%, p < 0.001), but accompanied cirrhosis was significantly fewer in younger subjects (17.4% vs. 63.2%, p < 0.001). Consistent with fewer cirrhotic patients in the younger group, the ICG-15 retention was lower (p = 0.0055) and the platelet counts tended to be higher (p = 0.087). There were no statistically significant differences in the remaining parameters between these two groups.

**Table 1:** Demographic data in relation to age of the training cohort HCC patients undergoing surgical resection

	**All patients**	**Age ≦40**	**Age >40**	p*
	**(n = 61)**	**(n = 23)**	**(n = 38)**	
**Patient demographics**				
Age (years) (median; 25 and 75 percentiles) (range)	47.5; 36.5-60.2 (26-75.7)	35.5; 30.8-37.0 (26-39.5)	56.8; 50.6-64.3 (40.8-75.7)	<0.001
Sex (M:F)	54:7	19:4	35:3	0.409
Albumin (g/dL) (median; 25 and 75 percentiles)	4.0; 3.8-4.4	4.2; 3.9-4.4	4.0; 3.7-4.3	0.129
Total bilirubin (mg/dL) (median; 25 and 75 percentiles)	0.80; 0.6-1.2	0.7; 0.5-1.1	0.9; 0.6-1.2	0.688
ALT (U/L) (median; 25 and 75 percentiles)	41.0; 30.5-57.0	46.0; 35.0-61.0	38.0; 29.8-57.0	0.946
AST (U/L) (median; 25 and 75 percentiles)	40.0; 27.0-66.0	42.0; 27.0-65.0	39.5; 27.0-67.5	0.871
Platelet (/mm3) (median; 25 and 75 percentiles)	182000; 139000-224500	204000; 171000-264000	167000; 131750-2017250	0.087
ICG-15R (%) (retention rate) (median; 25 and 75 percentiles)	8; 5.3-12.8	6; 4-8	10; 6-14.3	0.0055
Child-Pugh A/B (%)	59/2 (96.7%/3.3%)	22/1 (95.7%/4.4%)	37/1 (97.4%/2.6%)	1.000
**Tumor characteristics**				
Tumor size (cm) (median; 25 and 75 percentiles)	4.8; 3.4-8.7	5.0; 4.0-11.0	4.4; 3.2-6.4	0.523
Multinodularity (%)	28 (45.9%)	9 (39.1%)	19 (50.0%)	0.440
AFP (ng/ml) (median; 25 and 75 percentiles)	190; 15-1541	316; 18.4-5499	169.5; 12.9-728.5	0.742
Macroscopic venous invasion (%)	18 (29.5%)	14 (60.9%)	4 (10.5%)	<0.001
Daughter nodule (%)	32 (62.8%)	11 (55.0%)	21 (67.7%)	0.389
**Histopathological findings**				
Cirrhosis in non-tumor part (yes/no) (%)	33/28 (54.1%/45.9%)	4/19 (17.4%/82.6%)	24/14 (63.2%/36.8%)	<0.001
Edmondson grading (I or II/ III or IV) (%)	33/24 (57.9%/42.1%)	11/12 (47.8%/52.2%)	22/12 (64.7%/35.3%)	0.276
Microscopic venous invasion (%)	43 (70.5%)	17 (73.9%)	26 (68.4%)	0.775

ICG-15R: indocyanine green retention rate at 15 minutes;

*p value: comparison between younger and elderly HCC patients.

### Molecular signatures of yHCCs

**Figure 1:**
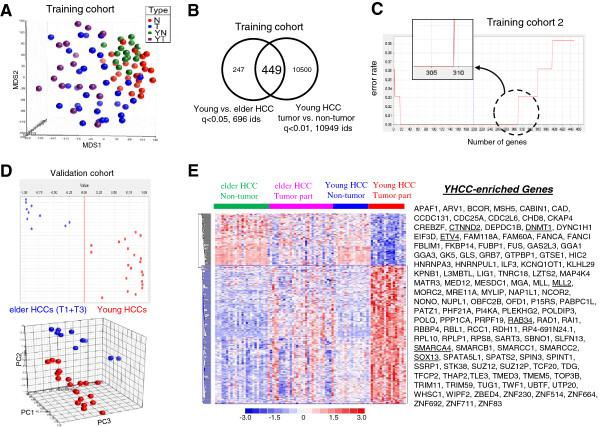
**Distinct gene expression patterns in HCC from young or elderly patients. (A)** A multidimensional scaling (MDS) plot using the whole transcriptome illustrates the mRNA profiles of normal and cancerous tissues. **(B)** A Venn diagram summarizing genes differentially expressed between the HCC tumor tissues of young and elderly patients, or between tumor and non-tumor yHCC samples. **(C)** Advanced signature training using a machine learning strategy and an independent testing elderly HCC data set. When probe sets were ranked by signal-to-noise ratios (weights), the top 309 features was the largest panel to give the lowest error rate (i.e., a best classification effect; *upper panel*). **(D)** The discrimination ability of the 309-probeset signature on the validation data set. The prediction strength plot (upper) and the PCA plot (lower) show the discriminating power of the identified 309 probe sets in separating young and elderly patients in the validation cohort. **(E)** A heat map shows the 309 probes sets differentiating young and elderly HCCs in the training data set 1, as well as discriminating tumor and non-tumor tissues. Columns represent tumor samples; rows represent probe sets. Genes in red: increased; in blue: decreased. Genes underlined: discussed in the text.

Data analysis steps were summarized in Additional file 1: Figure S1 online. To explore the molecular mechanisms governing the diverse clinical behaviors of the different HCCs, we delineated gene expression profiles of 48 primary HCC samples, as well as those of 39 non-cancerous tissues, from the above 61 patients as a training data set. A multidimensional scaling (MDS) plot using the whole transcriptome showed that the mRNA profiles of normal and cancerous tissues were different, while tumors of different age groups were similar (Figure 1A). We compared tumor samples to non-tumor counterparts for minimizing stromal and myometrial contamination. A total of 449 probe sets were differentially expressed between young and elderly HCCs (positive false discovery rate (pFDR) q < 0.05), as well as between tumor and non-tumor tissues of yHCC patients (Figure 1B).

The discrimination ability of these 449 probe sets were further trained by performing supervised machine learning that combined weighted voting algorithm and leave-one-out cross validation (LOOCV) [16], on the 2nd external data set (downloaded from the Expression Project for Oncology (expO)). An error rate of 9.4% (2 out of 16 yHCCs and 1 out of 16 elderly HCCs in the validation set; P < 0.001 by permutation test) was found (Figure 1C). The top 309 features (ranked by the weighted value of each probe set [16]) form a largest panel to have the best discrimination ability than that of the 449-probeset signature (error rate 0 vs. 9.4%; Figure 1C, upper panel). The discrimination ability of these 309 probe sets was evaluated on an independent testing data set that included another 21 yHCCs and 10 Taiwanese elderly HCCs (4 were at T1 stage and the remaining 6 were at T3 stage by 6th edition American Joint Committee on Cancer (AJCC)/International Union Against Cancer (UICC) staging system [17,18]). Prediction strength (PS; Figure 1D, upper) and principle component analysis (PCA; Figure 1D, lower) plots showed that these 309 probe sets distinguished young and elderly HCCs well.

The distribution of these 309 probe sets among sample groups were examined by hierarchical clustering. The differences in gene expression profiles between elderly and yHCC were more striking in tumor parts as compared to those in non-tumor parts (Figure 1E). A heat map for these genes indicated the unique gene expression levels in yHCC, with 225 probe sets being predominantly up in yHCCs (Table 2) while another 84 being down (Figure 1E). Many of yHCC-enriched genes, such as CTNND2 (delta 2 catenin), RAB34 (a member of the RAS oncogene family), SOX13 (SRY (sex determining region Y)-box 13), ETV4 (ets variant gene 4), DNMT1 (DNA cytosine-5-methyltransferase 1), TLE3 (transducin-like enhancer of split 3), MLL (myeloid/lymphoid or mixed-lineage leukemia), and MLL2, have been associated with tumor malignancy and poor patient outcomes in HCC or other cancers (Figure 1E, underlined). These consistent findings support the reliability of our gene list. Genes down-regulated in yHCC (i.e. more abundant in elderly HCCs) are shown in Additional file 2: Table S1.

**Table 2:** ESC genes overexpressed in yHCC patients (q < 0.05, Young HCC vs. elder HCC)

**Probe set ID**	**UniGene ID**	**Gene title**	**Gene symbol**	**Location**	**Folds**
219010_at	Hs.518997	chromosome 1 open reading frame 10	C1orf106	chr1q32.1	3.05
202715_at	Hs.377010	carbamoyl-phosphate synthetase 2, aspartate transcarbamylase, and dihydroorotase	CAD	chr2p22-p21	1.68
1555772_a_at	Hs.437705	cell division cycle 25 homolog A (S. pombe)	*CDC25A	chr3p21	1.89
226980_at	Hs.482233	DEP domain containing 1B	DEPDC1B	chr5q12.1	2.08
201697_s_at	Hs.202672	DNA (cytosine-5-)-methyltransferase 1	*DNMT1	chr19p13.2	1.74
229115_at	Hs.649497	dynein, cytoplasmic 1, heavy chain 1	*DYNC1H1	chr14q32.3-qter	1.78
200005_at	Hs.55682	eukaryotic translation initiation factor 3, subunit D	EIF3D	chr22q13.1	1.55
1554576_a_at	Hs.434059	ets variant gene 4 (E1A enhancer binding protein, E1AF)	ETV4	chr17q21	2.02
220060_s_at		family with sequence similarity 222, member B	FAM222B	chr17q11.2	1.56
213008_at	Hs.513126	Fanconi anemia, complementation group I	*FANCI	chr15q26.1	1.78
219390_at	Hs.571333	FK506 binding protein 14, 22 kDa	FKBP14	chr7p15.1	1.72
223079_s_at	Hs.116448	glutaminase	GLS	chr2q32-q34	2.26
215942_s_at	Hs.386189	G-2 and S-phase expressed 1	*GTSE1	chr22q13.2-q13.3	1.94
242890_at	Hs.655830	Helicase, lymphoid-specific	*HELLS	chr10q24.2	2.47
212966_at	Hs.632767	hypermethylated in cancer 2	*HIC2	chr22q11.21	2.71
208930_s_at	Hs.465885	interleukin enhancer binding factor 3, 90 kDa	*ILF3	chr19p13.2	2.32
208974_x_at	Hs.532793	karyopherin (importin) beta 1	KPNB1	chr17q21.32	1.82
202726_at	Hs.1770	ligase I, DNA, ATP-dependent	*LIG1	chr19q13.2-q13.3	1.82
65588_at	Hs.400876	lncRNA LOC388796	LOC388796	65588_at	1.68
224473_x_at	Hs.523221	leucine zipper, putative tumor suppressor 2	*LZTS2	chr10q24	1.61
64432_at	Hs.333120	MAPKAPK5 antisense RNA 1	MAPKAPK5-AS1	chr12q24.12	1.61
242260_at	Hs.268939	Matrin 3	MATR3	chr5q31.2	2.35
235409_at	Hs.187569	MAX gene associated	MGA	chr15q14	1.79
228097_at	Hs.484738	myosin regulatory light chain interacting protein	MYLIP	chr6p23-p22.3	1.64
208752_x_at	Hs.524599	nucleosome assembly protein 1-like 1	NAP1L1	chr12q21.2	1.66
214107_x_at	Hs.740414	aminopeptidase puromycin sensitive	NPEPPS	chr17q21	1.85
200057_s_at	Hs.533282	non-POU domain containing, octamer-binding	*NONO	chrXq13.1	1.52
228566_at	Hs.464912	Cyclin-dependent kinase 2B-inhibitor-related protein	P15RS	chr18q12.2	1.51
203103_s_at	Hs.502705	PRP19/PSO4 pre-mRNA processing factor 19 homolog (S. cerevisiae)	*PRPF19	chr11q12.2	1.49
1555630_a_at	Hs.301853	RAB34, member RAS oncogene family	RAB34	chr17q11.2	3.14
206499_s_at	Hs.469723	regulator of chromosome condensation 1	*RCC1	chr1p36.1	1.59
200858_s_at	Hs.512675	ribosomal protein S8	RPS8	chr1p34.1-p32	1.36
209127_s_at	Hs.584842	squamous cell carcinoma antigen recognized by T cells 3	SART3	chr12q24.1	2.03
214728_x_at	Hs.327527	SWI/SNF related, matrix associated, actin dependent regulator of chromatin, a4	*SMARCA4	chr19p13.2	1.61
201072_s_at	Hs.476179	SWI/SNF related, matrix associated, actin dependent regulator of chromatin, c1	SMARCC1	chr3p23-p21	1.89
228990_at	Hs.632377	small nucleolar RNA host gene 12 (non-protein coding)	SNHG12	chr1p35.3	1.86
218324_s_at	Hs.654826	spermatogenesis associated, serine-rich 2	SPATS2	chr12q13.12	1.84
202826_at	Hs.233950	serine peptidase inhibitor, Kunitz type 1	SPINT1 (HAI-1)	chr15q15.1	3.36
200956_s_at	Hs.523680	structure specific recognition protein 1	*SSRP1	chr11q12	1.78
207627_s_at	Hs.48849	transcription factor CP2	TFCP2	chr12q13	1.66
212770_at	Hs.709205	transducin-like enhancer of split 3 (E(sp1) homolog, Drosophila)	TLE3	chr15q22	1.51
208837_at	Hs.513058	transmembrane emp24 protein transport domain containing 3	TMED3	chr15q24-q25	2.77
238797_at	Hs.13543	tripartite motif-containing 11	TRIM11	chr1q42.13	1.51
235476_at	Hs.212957	tripartite motif-containing 59	TRIM59	chr3q26.1	2.00
209053_s_at	Hs.113876	Wolf-Hirschhorn syndrome candidate 1	WHSC1	chr4p16.3	1.97
204799_at	Hs.475208	zinc finger, BED-type containing 4	ZBED4	chr22q13.33	1.72
228988_at	Hs.326801	zinc finger protein 711	ZNF711	chrXq21.1-q21.2	3.40

*Genes discussed in the text.

### Coordinated functional module changes in yHCCs

**Figure 2:**
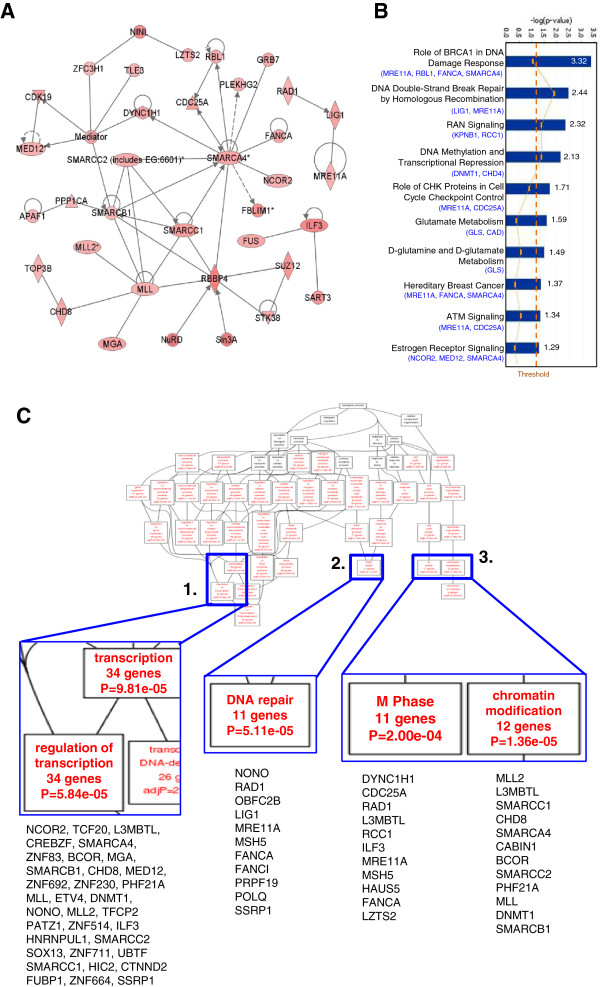
**Interaction network and gene set enrichment analyses as frameworks for interpreting yHCC biology. (A)** A genetic network composed of multiple yHCC genes. This network is displayed graphically as nodes (gene products) and edges (biological relationships between nodes) mapped by the Ingenuity Pathway Analysis (IPA) tool. The intensity of the node color indicates the degree of upregulation. **(B)** Canonical pathway analysis. Genes that are more abundant in yHCC were subjected to IPA search. **(C)** Altered biological modules in yHCCs*.* 282 probe sets that are more abundant in yHCC were subjected to Gene Ontology database search. The number of genes, gene symbols, and *p* values for each category that are significantly enriched are listed (*p* < 0.05).

To understand how genes enriched in yHCC are related to each other, as well as to spot the more critical yHCC genes, we performed systems biology analysis. A major genetic network contains known cancer-related or pro-proliferating genes, including CDC25A, CDK19, FUS (fused in sarcoma), TLE3, and ILF3 (interleukin enhancer binding factor 3) was formed (Figure 2A). Central to the network, there were hub genes (genes with higher connectivity to others), including MLL, SMARCA4, SMARCB1, SMARCC1, and RBBP4 (retinoblastoma binding protein 4) (Figure 2A).

To understand better how gene expression profiles correlate with pathogenesis and tumor phenotypes, signature probe sets were subjected into canonical pathways and functional group analysis using the Ingenuity Pathway Analysis (IPA) and Gene Ontology (GO) databases, respectively. The most significant canonical pathway mapped is the “BRCA1 in DNA Damage Response” pathway (Figure 2B). Other predominant pathways were DNA double-strand break repair, DNA methylation and transcriptional repression and ATM Signaling (Figure 2B). Consistent with the unique expression profile of yHCCs, the genes involved in the regulation of transcription were enriched in yHCCs (*p* = 5.84*10e-05; Figure 2C, panel 1). Genes involved in chromatin modification are also unique in yHCCs (*p* = 1.36*10e-5; Figure 2C, panel 3). Other related predominant GO processes included those pertaining to DNA repair (*p* = 5.11*10e-5) and M phase cell cycle (*p* = 2.00*10e-4) (Figure 2C, panels 2–3).

### Increased embryonic stem cell (ESC) traits in HCCs, especially those from young patients

**Figure 3:**
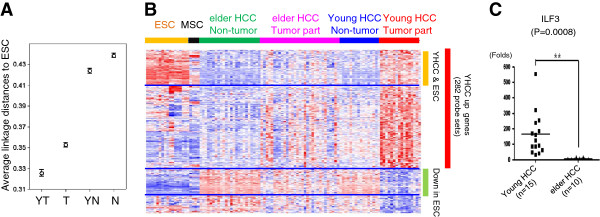
**ESC traits in yHCCs. (A)** Relationships between ESC, HCCs of different ages, and non-tumor tissues. Average linkage distances between tissues and ESC were calculated using the filtered 309 probe sets. The confidence limits as shown represent the standard error. YT and T: cancerous HCC samples from young and elderly patients, respectively. YN and N: non-tumor samples. **(B)** A heat map showing shared genes between yHCC and ESC. **(C)** Validation of ILF3 array data by real-time RT-PCR. The mean expression levels of the target genes were compared to the GAPDH control. **: *p* < 0.01 by *t*-test.

Stemness genes are known to contribute largely in tumorigenesis and disease progression [13,14]. For narrowing down key genes and obtaining more insights in yHCC pathogenesis, the above 309 probe sets were used to compare the relationships between HCCs and ESC. Transcriptome distances were measured by calculating the average linkage distances. Compared with non-tumor tissues, HCCs of different age categories were closer to ESCs (Figure 3A), suggesting the re-expression of ESC genes is a characteristic feature during tumorigenesis. The closest correlation between ESC and yHCC was observed, indicating the level of ESC gene re-expression was inversely correlated with patient age (Figure 3A).

The distributions of these 309 probe sets among sample groups were shown using a heat map (Figure 3B). Among genes enriched in yHCC, a subgroup of genes was also abundant in stem cells, especially in ESC (Figure 3B). Table 2 shows ESC genes overexpressed in YHCC patients. Among them, 9 genes were involved in cell cycle control (CDC25A, DYNC1H1, FANCI, GTSE1, HELLS, ILF3, LIG1, LZTS2, and RCC1; *p* = 1.3*10e-3, gene enrichment analysis was done based on the GO database), 5 genes in DNA repair (FANCI, PRPF19, LIG1, NONO, and SSRP1; *p* = 8.3*10e-3), and 2 genes in blastocyst growth (PRPF19 and SMARCA4; *p* = .031) (Table 2, genes with asterisks). Intriguingly, ILF3 is among the ‘Core 9’ ESC transcription regulators that were highly expressed in poorly differentiated breast cancers, glioblastomas, and bladder carcinomas (13). The differential expression of ILF3 between young and elderly HCCs was verified by RT-qPCR (Figure 3C).

### Decreasing hepatic differentiation program in yHCCs and during disease progression in elderly HCCs

**Figure 4:**
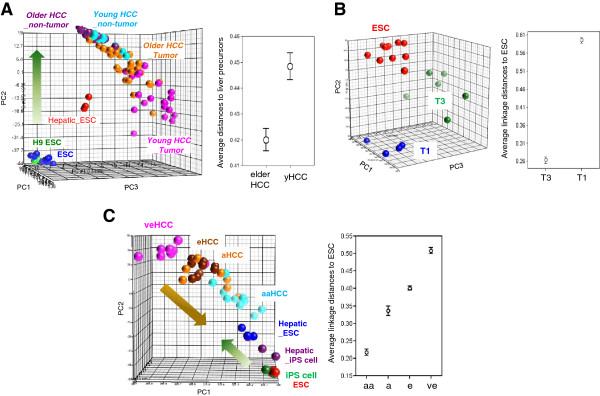
**Decreased hepatogenesis characters in yHCCs but increased ESC traits in advanced HCCs. (A)** Forfeiting of liver differentiation program in yHCCs. (*Left*) A PCA plot using genes differentiating the H9 ESCs and day 20 hepatic differentiated progenies (q < 10^-4^). The transcriptome drift directions during hepatic differentiation are indicated by an arrow. Hepatic_ESC: day 20 (d20) hepatic differentiated progenies. (*right*) Relationships between liver precursors and HCCs of different age groups. **(B)** Relationships between ESC and HCC of different histopathological stages. (*Left*) A PCA plot based on 977 probe sets genes distinguishing early (T1) and late (T3) HCCs from elderly patients. (*Right*) Relationships between ESC and T1/T3 HCCs. **(C)** Dedifferentiation-like transcriptome reprogramming during the progression of HCV-related HCC. (*Left*) A PCA plot using genes differentiating very early (ve) and advanced (a) HCC (q < 0.01, 1700 probe sets). The orange arrow represents the transcriptome drift direction during HCC progression. eHCC, early HCC; aHCC, advanced HCC; Hepatic_iPS cells: day 20 hepatic differentiated progenies of iPS cells. (*Right*) Relationships between ESC and HCC at different clinical stages.

We hypothesized that yHCCs also forfeited genes associated with liver differentiation and thereby were more dedifferentiated and malignant. Liver precursor characteristics were examined in the yHCC samples by comparing the relationships between HCC subgroups and liver progenitor cells (derived from the H9 ESC line [19]). An inverse correlation between the hepatogenesis process with patient ages was observed (Figure 4A, left panel; the direction of ESC hepatogenesis is indicated by a green arrow). Such impressions were strengthened by calculating the transcriptome distances between the sample groups (Figure 4A, right panel). Among the 309 yHCC genes, 15 genes were more abundant in differentiated liver progenitor cells (day 20; Additional file 3: Figure S2 online). These 15 genes, which are also downregulated in yHCCs, hold the potentials of being novel tumor suppressor genes in yHCCs.

The above observation inspired us to hypothesize further that the forfeiting of hepatogenesis traits may have also occurred during disease progression in HCCs of the same age group. We examined the associations between ESC gene patterns and clinical stage. Early (T1) and late (T3) HCCs [18] used in the validation cohort were applied to compare the relationships with ES cells and the advanced T3 cases were closer to ES cells (Figure 4B). Such relationships were validated by evaluating another independent serum anti-HCV positive HCC data set [20]. This data set included four neoplastic stages (very early HCC to very advanced metastatic tumors) from patients with HCV infection [20]. When the relationships between the different pathological HCC subgroups and pluripotent stem cells (including ESCs and induced pluripotent stem cells (iPS cells) [19]) were compared, an increased stemness that accurately reflected the pathological progression of the disease was again observed (Figure 4C). A dedifferentiation-like transcriptome drift (indicated by an orange arrow, Figure 4C) was anti-correlated with the hepatic differentiation program of pluripotent stem cells (indicated by a green arrow), indicating a dedifferentiation status during the progression of HCV-related HCC.

## Discussion

This study explored the gene expression profile of yHCC. We found the age difference between HCC patients is mirrored in their gene expression profiles. A similar observation has been reported for other cancers: there was a clear segregation of the pediatric and adult germ cell tumors [21], and pediatric glioblastomas also have a characteristic transcriptome profile different from that of adult tumors [22,23]. The outcomes of melanoma in the younger and the elderly populations were also different and these 2 patient groups express distinct microRNA profiles [24]. Thus, age difference between patients with the same disease can be mirrored in their gene expression profiles. Patients of different ages but with the same tumor should be treated in different ways.

Gender disparity is a well known phenotype in HCC, and animal studies suggest that it may be due to the stimulatory effects of androgen and the protective effects of estrogen (see reviews [25,26]). Estrogen can protect hepatocytes from malignant transformation [27]. Intriguingly, both the androgen receptor (AR) and estrogen receptor 1 (ESR1) sex hormone receptors are down-regulated in yHCCs (Additional file 2: Table S1 & not shown). Genes involved in estrogen receptor signaling are also enriched in the yHCC signature (Figure 2B). Since all of our yHCC patients were sexually matured (the youngest case is a 26-year old female; Table 1), our data indicates an original and a unique pathogenesis mechanism in yHCCs.

HCC with stemness-related marker expression has recently been proposed to be a new and more aggressive subtype of HCC [28,29]. It is important that a suitable marker panel is developed to facilitate the diagnosis of this devastating HCC subtype. RT-qPCR analysis on elderly HCCs demonstrated that the high expression levels of 7 putative hepatic stem/progenitor cell biomarkers (including keratin 19 (K19), ABCG2, CD44, Nestin, CD133, EPCAM and OV6), is related to tumor angiogenesis and a poor prognosis for the HCC [15,28]. Recently, a stemness-related marker, CK19, was found well correlated with clinicopathologic features of tumor aggressiveness, vascular invasion, and poor differentiation in elderly HCCs [30]. No similar study has been addressed on yHCCs. Identifying genes involved in both cancer progression and cell dedifferentiation will offer another dimension to pathogenesis mechanisms, as well as providing novel therapeutic targets and prognosis markers. ILF3 (NF90) is one of the shared top genes between ESC and yHCC. LIF3 is among the ‘Core 9’ ESC genes highly re-expressed in advanced and poorly differentiated tumors [13] and is a prognostic factor in non-small cell lung cancer [31]. Another ESC gene overexpressed in yHCCs is DNMT1 and is responsible for the maintenance of DNA methylation patterns during replication. Inhibitors of this enzyme may potentially lead to DNA hypomethylation and re-expression of tumor suppressor genes [32]. Also, SOX13 contributes to control Wnt/TCF activity [33], crucial in HCC pathogenesis and cancer stem cell renewal [34]. Targeting these genes or pathways may restrain invasion by yHCC.

In addition to stemness genes, we also filtrated out 15 differentiation-related genes from in yHCCs. Eleven of these genes, including GSTK1 (glutathione S-transferase kappa 1) and SAR1B (SAR1 gene homolog B), are within the top 50 most down-regulated genes in yHCC patients (Additional file 2: Table S1; labeled with asterisks in Additional file 3: Figure S2). The repressed transcript levels and increased gene expression patterns during ESC hepatogenesis implied that these genes might function as novel tumor suppressor genes (TSGs). GSTK1 belongs to the glutathione S-transferase (GST) gene family that are critical for detoxification *via* conjugation of reduced glutathione (GSH) with numerous substrates such as pharmaceuticals and environmental pollutants [35]. GSTP1, another member of the GST family, has recently been identified to be a novel TSG for elderly HCCs, and the methylation frequency in GSTP1 is associated with HCC occurrence [36]. Roles of GSTK1 in yHCCs tumorigenesis and prognosis, as well as in ESC hepatogenesis, are awaited to be elucidated in the future.

## Conclusion

This study revealed the molecular makeup of yHCC and the link between ESC traits and HCC subtypes. Therefore, molecular mechanisms in elderly HCC patients cannot be simply extrapolated to younger patients. Our results also helped to identify transcriptional programs that can be used as potential therapeutic targets for various HCC subgroups.

## Methods

### Patient profiles and microarray expression data sets

Data analysis and RNA isolation details were summarized in Additional file 4: *Supplementary Materials and Methods* online. The diagnosis of all the HCC patients had been tissue-verified by pathological examination of the surgically removed HCC and neighboring liver tissue. All 44 young HCCs (≤40 years old at the diagnosis; 23 cases in the training set while another 21 in the validation cohort) were positive for serum hepatitis B surface antigen (HBsAg), but negative for antibodies to hepatitis C virus (anti-HCV). All 48 elderly (>40 years old; 38 in the training set while another 10 in the validation cohort) HCC patients enrolled were also serum HBsAg positive and anti-HCV negative. The HCC samples used in this study were the original tumors obtained from the first operations of patients. The current study complies with the Helsinki Declaration. Informed consents for taking small part of the resected HCC and the surrounding non-tumor liver specimens for study were obtained from patients. The tissue sample analysis was approved by the Institutional Review Board of Taipei Veterans General Hospital (VGHIRB No.: 97-09-17A), Taiwan.

Fresh HCC tissues and non-tumor counter parts that had been removed during surgery were snap frozen and kept in liquid nitrogen for RNA extraction. All array data were deposited into the NCBI Gene expression omnibus (GEO; http://www.ncbi.nlm.nih.gov/geo/) database [37] with the accession number GSE45436 (see Additional file 1: Figure S1; training set 1 GSE45267, training set 2 GSE45434, and validation set GSE45435).

The embryonic stem cell (ESC) array data had been published previously [38]. HCV (+) HCC array data were downloaded from the GEO database (accession number GSE6764) [20]. Array data of the induced pluripotent stem cells (iPS cells) and ESCs, as well as their hepatic differentiated progenies, were from GEO dataset GSE14897 [19]. The second batch of elderly HCCs of the training data set were downloaded from the Expression Project for Oncology (expO) of the International Genomics Consortium (http://www.intgen.org/, accession number GSE2109 in the GEO database).

## Abbreviations

HCC: Hepatocellular carcinoma; yHCC: Young HCC; ESC: Embryonic stem cells; HBV: Hepatitis B virus; HBsAg: Hepatitis B surface antigen; HCV: Hepatitis C virus; GEO: Gene expression omnibus; iPS: Induced pluripotent stem cells; FDR: False discovery rate; PCA: Principle component analysis; PS: Prediction strength; IPA: Ingenuity pathway analysis; GO: Gene Ontology; LOOCV: Leave-one-out-cross validation.

## Competing interests

The authors declare that they have no competing interests.

## Authors’ contributions

HW Wang and JC Wu conceived the study and identified its value to research. HW Wang and JC Wu designed the analysis approach. GY Chau, CW Su, and JC Wu collected tumor samples and carried out clinicopathologic feature analysis. HW Wang, SY Huang, TH Hsieh and CY Tung carried out the implementation of data analysis and wetlab experiments. HW Wang and JC Wu provided biological guidance during the analysis process. The manuscript was written by HW Wang and then revised by JC Wu. All authors read and approved the final manuscript.

## Supplementary Material

Additional file 1: Figure S1 A workflow summarizing experimental design and the filtration of yHCC genes. Click here for file

Additional file 2: Table S1 Top 50 genes down-regulated in yHCC patients (q < 0.05, Young HCC vs. elder HCC). Click here for file

Additional file 3: Figure S2 Differentiation-related yHCC genes. A Venn diagram illustrates that 83 ESC hepatogenesis-related yHCC genes are also present in the 309 yHCC genes. A heat map based on these 83 genes is shown. Array data of H9 ESC and differentiated liver precursor cells (day 20, d20) [19] were from GEO dataset GSE14897. *: genes discussed in the text. Click here for file

Additional file 4 Supplementary Materials and Methods. Click here for file
