# Supplementary material for: Forfeited hepatogenesis program and increased embryonic stem cell traits in young hepatocellular carcinoma (HCC) comparing to elderly HCC
Source: BMC Genomics. 2013 Oct 26;14:736. doi: 10.1186/1471-2164-14-736 (PMC3826595; doi:10.1186/1471-2164-14-736)
Supplement: Additional file 1: Figure S1 — A workflow summarizing experimental design and the filtration of yHCC genes. [file 1471-2164-14-736-S1.pptx]

## Slide 1
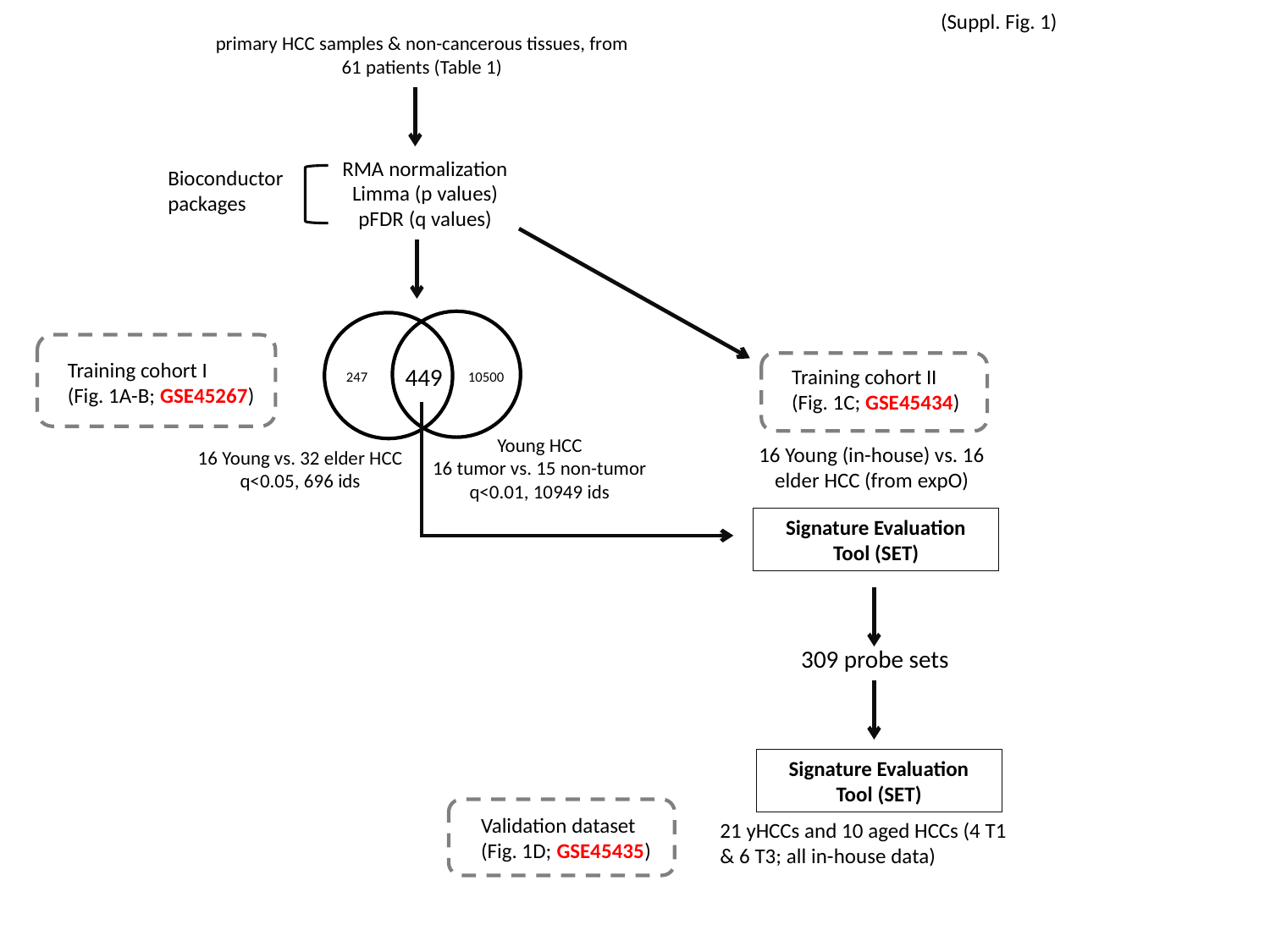

(Suppl. Fig. 1)
primary HCC samples & non-cancerous tissues, from 61 patients (Table 1)
RMA normalization
Limma (p values)
pFDR (q values)
Bioconductor
packages
Training cohort I
(Fig. 1A-B; GSE45267)
449
Training cohort II
(Fig. 1C; GSE45434)
247
10500
Young HCC
16 tumor vs. 15 non-tumor
q<0.01, 10949 ids
16 Young (in-house) vs. 16 elder HCC (from expO)
16 Young vs. 32 elder HCC
q<0.05, 696 ids
Signature Evaluation Tool (SET)
309 probe sets
Signature Evaluation Tool (SET)
Validation dataset
(Fig. 1D; GSE45435)
21 yHCCs and 10 aged HCCs (4 T1 & 6 T3; all in-house data)
