# Supplementary material for: Forfeited hepatogenesis program and increased embryonic stem cell traits in young hepatocellular carcinoma (HCC) comparing to elderly HCC
Source: BMC Genomics. 2013 Oct 26;14:736. doi: 10.1186/1471-2164-14-736 (PMC3826595; doi:10.1186/1471-2164-14-736)
Supplement: Additional file 3: Figure S2 — Differentiation-related yHCC genes. A Venn diagram illustrates that 83 ESC hepatogenesis-related yHCC genes are also present in the 309 yHCC genes. A heat map based on these 83 genes is shown. Array data of H9 ESC and differentiated liver precursor cells (day 20, d20) [19] were from GEO dataset GSE14897. *: genes discussed in the text. [file 1471-2164-14-736-S3.pptx]

## Slide 1
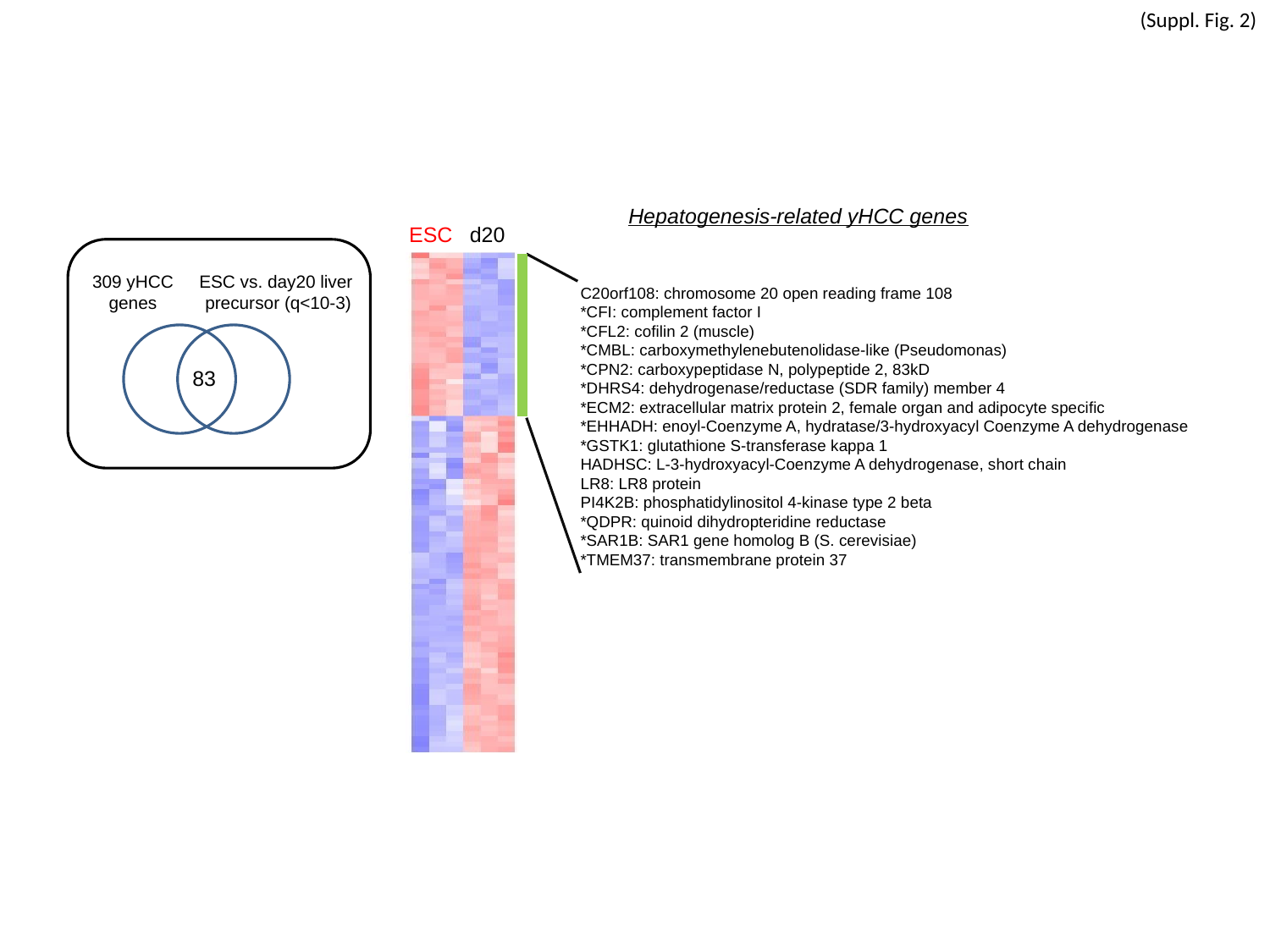

(Suppl. Fig. 2)
Hepatogenesis-related yHCC genes
ESC
d20
ESC vs. day20 liver
precursor (q<10-3)
309 yHCC
genes
C20orf108: chromosome 20 open reading frame 108
*CFI: complement factor I
*CFL2: cofilin 2 (muscle)
*CMBL: carboxymethylenebutenolidase-like (Pseudomonas)
*CPN2: carboxypeptidase N, polypeptide 2, 83kD
*DHRS4: dehydrogenase/reductase (SDR family) member 4
*ECM2: extracellular matrix protein 2, female organ and adipocyte specific
*EHHADH: enoyl-Coenzyme A, hydratase/3-hydroxyacyl Coenzyme A dehydrogenase
*GSTK1: glutathione S-transferase kappa 1
HADHSC: L-3-hydroxyacyl-Coenzyme A dehydrogenase, short chain
LR8: LR8 protein
PI4K2B: phosphatidylinositol 4-kinase type 2 beta
*QDPR: quinoid dihydropteridine reductase
*SAR1B: SAR1 gene homolog B (S. cerevisiae)
*TMEM37: transmembrane protein 37
83
