# Supplementary material for: Forfeited hepatogenesis program and increased embryonic stem cell traits in young hepatocellular carcinoma (HCC) comparing to elderly HCC
Source: BMC Genomics. 2013 Oct 26;14:736. doi: 10.1186/1471-2164-14-736 (PMC3826595; doi:10.1186/1471-2164-14-736)
Supplement: Additional file 4 — Supplementary Materials and Methods. [file 1471-2164-14-736-S4.docx]

***Additional file 4***

***Supplementary Materials and Methods***

***Array data analysis***

Snap frozen fresh cancerous tissues of the original tumors from 16 yHCCs and 32 elder HCC patients, as well as non-cancerous tissues from 15 yHCCs and 24 elder HCCs, were collected and then subjected into gene expression microarray analysis. Total RNA sample preparation, cRNA probe preparation, array hybridization and data analysis were done as described previously [[1](#_ENREF_1), [2](#_ENREF_2)]. Affymetrix^TM^ HG-U133 Plus 2.0 whole genome chips were used. RMA log expression units were calculated from Affymetrix GeneChip array data using the ‘affy’ package of the Bioconductor ([http://www.bioconductor.org](http://www.bioconductor.org/)) [[3](#_ENREF_3)] suite of software for the R statistical programming language ([http://www.r-project.org](http://www.r-project.org/)). The default RMA settings were used to background correct, normalize and summarize all expression values. Significant difference between sample groups was identified using the ‘*limma*’ package of the Bioconductor according to the method described by Storey & Tibshirani [4]. Briefly, a t-statistic was calculated as normal for each gene and a *p*-value then calculated using a modified permutation test [4]. To control the multiple testing errors, a false discovery rate (FDR) algorithm was then applied to these *p*-values to calculate a set of *q*-values: thresholds of the expected proportion of false positives, or false rejections of the null hypothesis. Heat maps were created by the dChip software (<http://www.dchip.org/>) [[4](#_ENREF_4)]. Principal component analysis (PCA) and multidimensional scaling (MDS) was performed by the Partek Genomics Suite (http://www.partek.com/) to provide a visual impression of how the various sample groups are related. Gene annotation was performed by the ArrayFusion web tool (<http://microarray.ym.edu.tw/tools/arrayfusion/>) [[5](#_ENREF_5)]. Gene Ontology (<http://www.geneontology.org/>) [[6](#_ENREF_6)] database search were performed by the WebGestalt interface (<http://bioinfo.vanderbilt.edu/webgestalt/>) (29).

To evaluate the discrimination power of the filtered genes, the uploaded genes are ranked according to the absolute values of corresponding signal-to-noise scores [[7](#_ENREF_7)] in a descending order. Genes are included into a signature one at a time based on the order of ranking. The error rate for each new signature is estimated by the weighted voting algorithm and LOOCV and can be monitored by an error rate distribution plot [[8](#_ENREF_8)]. Based on the error rate information, we then selected an appropriate composition for the discriminating genes with the lowest error rate. Once a signature is defined, the result of prediction strength (PS) analysis for each sample is shown. The PS values range from -1 to +1, where higher absolute values reflect stronger predictions [[8](#_ENREF_8)]. An overview of the results for samples in the different groups is then illustrated by a PS plot [[8](#_ENREF_8)].

The average linkage distance between samples is calculated by Pearson correlation subtracted from unity to provide bounded distances in the range (0, 2), as described in our previous studies [[1](#_ENREF_1), [9](#_ENREF_9)]. The distance between two groups of samples is calculated using the average linkage measure (the mean of all pair-wise distances (linkages) between members of the two groups concerned). The standard error of the average linkage distance between two groups (the standard deviation of pair-wise linkages divided by the square root of the number of linkages) is quoted when inter-group distances are compared in the text.

In order to obtain functional regulatory networks, the filtered features from the array analysis are subjected to Ingenuity Pathway analysis (IPA) software (<http://www.ingenuity.com/>) [[10](#_ENREF_10)]. The knowledge base behind IPA was built upon scientific evidence, manually curated from thousands of journal articles, textbooks, and other data sources. After a list of signature genes is uploaded, interaction among the focus genes and interaction among the interacting genes and molecules from the knowledge base are used to combine genes into networks according to their probability of having more focus genes than expected by chance. The term “network” in IPA is not the same as a biological or canonical pathway with a distinct function but a reflection of all interactions of a given protein as defined in the literature. Networks are scored on the basis of the number of uploaded signature genes they contain. The network score is based on the hypergeometric distribution and is calculated using a right-tailed Fisher’s exact test. The score is the negative log of this *p* value. The higher the score, the lower the probability of finding the observed number of uploaded signature genes in a given network by random chance [[9](#_ENREF_9)].

***RNA isolation and real-time quantitative polymerase chain reaction***

Total mRNA were extracted by RNeasy mini kit (Cat. 74106; Qiagen GmbH, Hilden, Germany) and 100 ng to 1μg of total RNA was used for reverse transcription using the SuperScript^TM^ III Reverse transcriptase kit (Invitrogen, Carlsbad, CA, USA) as directed by the manufacturer. For quantitative real-time PCR analysis, the human pre-messenger RNA sequences were obtained from the NCBI (National Center for Biotechnology Information) AceView program ([www.ncbi.nlm.nih.gov/AceView/](file:///C:\Users\hwwang\AppData\Local\Microsoft\Windows\Temporary%20Internet%20Files\Content.Outlook\2O4N1G69\www.ncbi.nlm.nih.gov\AceView\)). All primers were designed to cross introns as specified by the Primer3 website (<http://frodo.wi.mit.edu/cgi-bin/primer3/primer3_www.cgi/>) or Primer Express software (Applied Biosystems, Foster City, CA, USA), and thermodynamics and primer specificity analysis were performed by the Vector NTI suite (Invitrogen, USA) and the NCBI reverse e-PCR program (<http://www.ncbi.nlm.nih.gov/sutils/e-pcr/reverse.cgi/>). Real-time PCR reactions were performed using Maxima^TM^ SYBR Green qPCR Master Mix (Cat. K0222; Fermentas, Glen Burnie, Maryland, USA), and the specific products were detected and analyzed using a StepOne^TM^ sequence detector (Applied Biosystems, USA). The expression level of each gene was normalized to the expression level of Glyceraldehyde 3-phosphate dehydrogenase (GAPDH). All the primer sequences are as following: GAPDH: ggAgTCCACTggCgTCTTCA and TggTTCACACCCATgACgAA; ILF3: AgAggCgTgggCTCAAgTAC and CATCCACTTCgACCTCCATgA.

**References**

1. Wang HW, Trotter MW, Lagos D, Bourboulia D, Henderson S, Makinen T, Elliman S, Flanagan AM, Alitalo K, Boshoff C: **Kaposi sarcoma herpesvirus-induced cellular reprogramming contributes to the lymphatic endothelial gene expression in Kaposi sarcoma**. *Nat Genet* 2004, **36**(7):687-693.

2. Wang HW, Wu YH, Hsieh JY, Liang ML, Chao ME, Liu DJ, Hsu MT, Wong TT: **Pediatric primary central nervous system germ cell tumors of different prognosis groups show characteristic miRNome traits and chromosome copy number variations**. *BMC Genomics* 2010, **11**:132.

3. Reimers M, Carey VJ: **Bioconductor: an open source framework for bioinformatics and computational biology**. *Methods in enzymology* 2006, **411**:119-134.

4. Schadt EE, Li C, Ellis B, Wong WH: **Feature extraction and normalization algorithms for high-density oligonucleotide gene expression array data**. *Journal of cellular biochemistry Supplement* 2001, **Suppl 37**:120-125.

5. Yang TP, Chang TY, Lin CH, Hsu MT, Wang HW: **ArrayFusion: a web application for multi-dimensional analysis of CGH, SNP and microarray data**. *Bioinformatics* 2006, **22**(21):2697-2698.

6. Lomax J: **Get ready to GO! A biologist's guide to the Gene Ontology**. *Briefings in bioinformatics* 2005, **6**(3):298-304.

7. Ramaswamy S, Ross KN, Lander ES, Golub TR: **A molecular signature of metastasis in primary solid tumors**. *Nat Genet* 2003, **33**(1):49-54.

8. Jen CH, Yang TP, Tung CY, Su SH, Lin CH, Hsu MT, Wang HW: **Signature Evaluation Tool (SET): a Java-based tool to evaluate and visualize the sample discrimination abilities of gene expression signatures**. *BMC Bioinformatics* 2008, **9**(1):58.

9. Huang TS, Hsieh JY, Wu YH, Jen CH, Tsuang YH, Chiou SH, Partanen J, Anderson H, Jaatinen T, Yu YH *et al*: **Functional network reconstruction reveals somatic stemness genetic maps and dedifferentiation-like transcriptome reprogramming induced by GATA2**. *Stem Cells* 2008, **26**(5):1186-1201.

10. Thomas S, Bonchev D: **A survey of current software for network analysis in molecular biology**. *Hum Genomics* 2010, **4**(5):353-360.
